# Mechanistic constraint and evolutionary history matter for the origin of innovation in animal communication

**DOI:** 10.1093/beheco/arag044

**Published:** 2026-04-27

**Authors:** Terry J Ord

**Affiliations:** Evolution and Ecology Research Centre, and the School of Biological, Earth and Environmental Sciences, University of New South Wales, Kensington, NSW 2052, Australia

**Keywords:** adaptive peak, animal behavior, functional redundancy, historical contingency, many-to-one form-to-function, natural selection, novelty, sexual selection

## Abstract

Numerous phylogenetic comparative studies have attempted to explain differences in species phenotypes as a function of present-day social or physical environments. In some cases, these investigations have also shown dramatic convergences among distantly related species, illustrating how common selection pressures can produce repeated adaptation. In other cases, distantly related species exhibit phenotypes that appear quite different, and despite putative similarities in selection. Understanding why this is the case is central to our broader knowledge of how evolution operates in nature, and what accounts for the diversity we see in the natural world. Species responding differently to common selection pressures likely traces back to mechanistic constraints and evolutionary starting points bounding the direction of adaptation. These are common themes in evolutionary biology and there is a long history dating back to Lorenz and Tinbergen of considering “proximate” and “ultimate” factors for interpreting present-day behavior. Yet, integration of mechanism and history is still often missing from behavioral ecology. This might reflect the reasonable assumption that behavior is, for the most part, shaped through plasticity or adaptive evolution in response to conditions existing in present-day environments. Yet this assumption fails to explain why species in similar environments so often differ in behavior. Only when behavior is placed into its broader phylogenetic context and explored through the lens of “paths-of-least-resistance” does adaptive innovation even become apparent. Iconic case studies including the sword of swordtails, the song of Darwin finches and the dewlap of anole lizards are examined through this lens to reveal hidden trajectories of adaptation that have led to innovation and diversity.

## Teaser

Both physiology and historical events matter for the evolution of new forms of animal communication. This review showcases what we currently know and how a shift in research perspective may lead to a useful re-evaluation of our understanding of the adaptive trajectory of signal evolution

## Introduction

When attempting to explain the way animals communicate with one another, a communication biologist might start by reflecting on 2 foundational factors: the function of the signal and how the environment impacts that function. We know signal form follows function as a classic first principle (Marler 1967; Morton 1977). For example, aggressive calls in birds and mammals are usually harsh, broad band sounds concentrated towards low frequencies designed to be highly localizable and convey reliable cues on a signaler's body size (which is important in winning fights; Morton 1977). In contrast, alarm calls or “seet” calls in birds are narrow, high frequency sounds designed to alert nearby conspecifics but are difficult for a distant predator to detect and localize (Marler 1955), and the same call design has evolved independently as an alarm in squirrels (Greene and Meagher 1998; see also Wilson and Hare 2004). Both examples illustrate how the range of forms (or signal designs) suitable for accurately (or honestly) fulfilling a function is further whittled down to those readily perceived by intended receivers in the environments and over the distances typical for signal transmission (Alexander 1962; Morton 1975; Wiley and Richards 1978; Brenowitz 1986).

Both signal function and environmental-receiver influences can be thoroughly investigated through observational and experimental study of a given species, and there is a long history of exactly this type of research (reviewed by Marler 1967; Brenowitz 1986; Hasson 1991; McGregor 1992; Holldobler 1999; Gerhardt and Huber 2002; Uetz and Roberts 2002; Bradbury and Vehrencamp 2011; Wiley 2015; see also O’Hanlon et al. 2025). A communication biologist might then wish to explain the diversity across species in the way animals communicate, and quite reasonably continues to do so through the perspective of adaptation as it relates to present-day function and environmental-receiver context. There are numerous studies comparing the social and physical environments across species and how those might account for differences in behavior (or convergences; see following paragraph). For example, as competitive pressure for territories and mates increases across closely-related species, the signals used in mediating those interactions often become progressively more complex to improve opponent assessment or mate attraction (Catchpole 1980; Catchpole and McGregor 1985; Shutler and Weatherhead 1990; Ryan and Rand 1993; Hill 1994; Ord et al. 2001; Price and Lanyon 2004; Chen et al. 2012; see also Irwin 1990). We also know the visual environment determines the types of colors and patterns that can be readily detected or perceived by receivers, and this leads to predictable diversity in ornamentation across species living in different habitats (eg Marchetti 1993; Fleishman 2000; Seehausen et al. 2008; Hulse et al. 2020; Yao and Ord 2025). The physical environment impacts other forms of communication as well (Morton 1975), for example resulting in differentiation in the design of acoustic signals (Irwin et al. 2001; Derryberry 2009; Tobias et al. 2010), movement displays (Ord et al 2010; Ligon et al. 2018) and olfactory signals (Baeckens et al. 2018) as a function of how easily receivers detect those signals in a given environment.

We also expect adaptive convergence whenever distantly-related species live in similar environments or are otherwise exposed to similar selection pressures (eg Endler 1982; Blackledge and Gillespie 2004; Hoekstra et al. 2006; Rosenblum et al. 2010; Mahler et al. 2013; Barshai et al. 2021). Despite animal communication clearly being influenced by the environment, there are surprisingly few robust cases of signal convergence (reviewed by Ord and Summers 2015; see also Anderson et al. 2024). Whether this reflects a lack of research or biological reality is unclear, but convergence in animal communication certainly occurs to some degree. While the consistent design of the “seet” alarm call among bird species has yet to be formally demonstrated as not reflecting homology (see also Jurisevic and Sanderson 1994), it seems clear the design of avian “seet” calls (Marler 1955) and certain squirrel alarm calls (Greene and Meagher 1998) have originated independently (even if a formal phylogenetic investigation has yet to be conducted). There is also evidence these calls in birds and squirrels are not only similar in design, but in function and context as well (both given in response to raptors; Greene and Meagher 1998). Other notable convergences include the courtship calls of certain Northern American and Asian crickets that are so similar in design to elicit comparable responses from heterospecific mates (Henry et al. 1999). Common designs in the territorial advertisement displays performed by Caribbean anole lizards have evolved independently on at least 2 islands where species communicate in similar light environments (Ord et al. 2013). Foot-flagging threat displays in frogs have convergently evolved at least 6 times in species living in noisy environments (Anderson et al. 2024). There are many broad convergences in signal behavior for coping with environmental noise across major taxonomic groups as well (reviewed by Ord et al. 2021).

**Figure 1:**
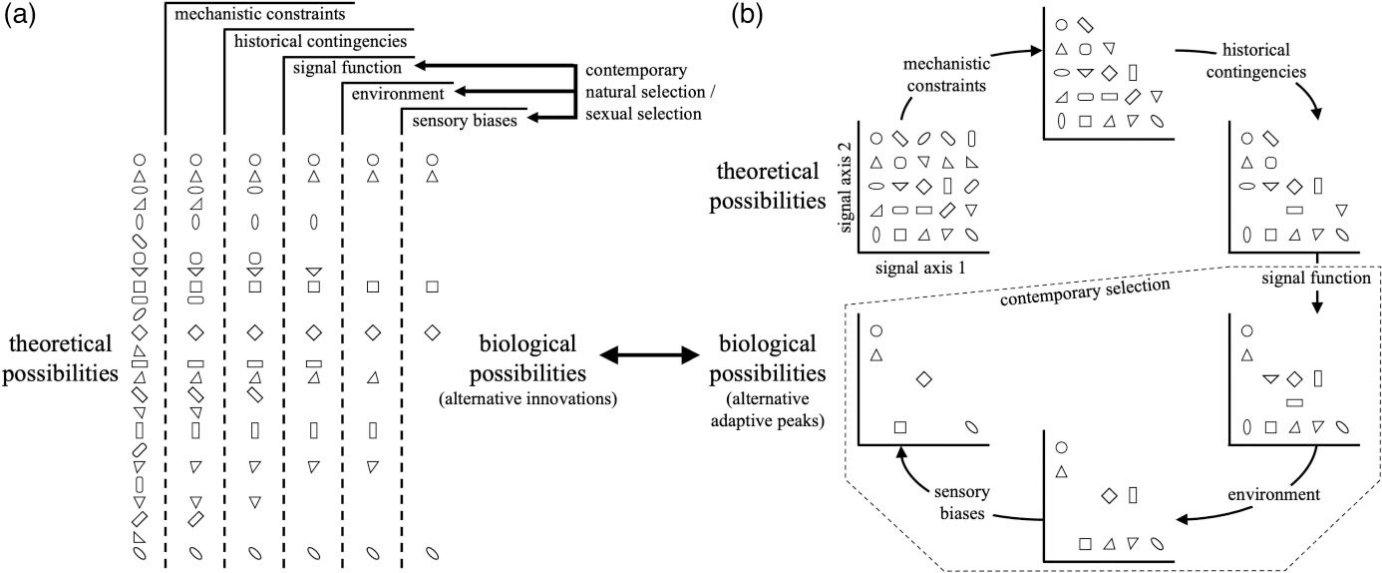
How mechanistic constraints and historical contingencies impact adaptive outcomes to produce innovations that reveal alternative adaptive peaks. Physiological and morphological constraints, coupled with the contingent nature of the evolution process (how it builds on what has gone before), filters an effectively infinite pool of theoretically possible options. These constraint and contingency filters subsequently dictate the possibilities that might arise in response to present-day selection (from the physical or social environments taxa occupy today). This paradigm can be viewed as whittling down a) a categorical set of signal types, components or behaviors, or b) variation along continuous multidimensional signal axes. The paradigm begins with mechanistic constraints defining what is and is not possible. Of the mechanistically available options, only a subset will eventually arise through random mutation, epistasis and depending on a lineage's phenotypic starting point. These contingent options are further filtered by contemporary natural selection or sexual selection (or both) to those capable of fulfilling a specific signal function. All signals must then travel through the environment before reaching receivers, and some options will be better than others in retaining functional integrity, adding a further selection filter into the mix. Finally, of these efficiency options, some will more readily stimulate receiver sensory or cognitive systems. These remaining options are the true (biological) possibilities that could evolve. While there is extensive research on how differences (or similarities) in signal function, environment and receiver systems might lead to adaptive differentiation (or convergence) among taxa, it misses the principal, first-order filters of mechanistic constraint and historical contingency. Why is this a potential issue? Consider a situation where different taxa are exposed to identical contemporary selection pressures: a signal must fulfill a specific function, must retain its integrity as it travels through the same environment, and must stimulate receivers with identical sensory and cognitive biases. Yet, each lineage comes with unique physiological or morphological constraints, or from different phenotypic starting points, (or both) which corrals adaptive evolution along novel paths-of-least-resistance. The resulting outcomes represent alternative adaptive solutions to the same selective challenge, or functional redundancy. This diversity reflects adaptive innovation and illuminates the range of adaptive peaks possible for a common selection pressure. Explicitly considering mechanistic constraints and historical contingencies therefore provides better resolution of the adaptive process while also revealing the origins of behavioral diversity across species.

Both focused study on single species and comparative study across species have broadened our understanding of animal communication tremendously, especially for identifying the factors that influence its evolution (eg reviewed by Bradbury and Vehrencamp 2011). However, the implicit or explicit emphasis on contemporary outcomes of natural selection or sexual selection (or their interaction) potentially misses a richer story offering new insights on how and why animal communication has evolved the way it has in present-day species. Specifically, animal behavior is shaped by mechanistic constraints associated with physiology and morphology, coupled with the contingent nature of the adaptive process building on historic starting points (not to mention its reliance on random mutation; Fig. 1). Yet, these constraints and contingencies are often missing from our current thinking around the evolution of animal communication (eg see Podos 2022; Anderson et al. 2024; MacGillavry 2025), despite early emphasis of their importance in the origins of behavior made decades ago (Lorenz 1941; Tinbergen 1963; see also Brooks and McLennan 1991; Ryan and Rand 1993; Martins 1996). Reestablishing this viewpoint in current research will necessarily require moving beyond the traditional phylogenetic comparative approach of simply correcting for the “confounding effect” of phylogeny (eg Rohlf 2006; O’Hanlon et al. 2025), to 1 leveraging the phylogeny itself to explicitly identify how evolutionary history has bound the expression of present-day behavior (eg Ord 2012; Miles and Fuxjager 2019; Miles et al. 2020; Ord et al. 2023).

The examples cited throughout the text above offer cases of where adaptive explanations of behavior based on present-day biotic or abiotic environments have advanced our understanding in numerous ways, and often without any need to evoke the mechanistic basis of behavior. In the sections that follow below, my objective is not to refute the value of these insights or claim current practice is somehow flawed. Rather, I will present the case of how explicit consideration of mechanistic constraints and historical starting points provide an extra-dimension to our knowledge (Fig. 1) and should subsequently be reinstated in our intellectual toolkit when interpreting why animals behave the way they do (Lorenz 1941; Tinbergen 1963; Brooks and McLennan 1991; Ryan and Rand 1993; Martins 1996).

## Constraints, contingencies and paths-of-least-resistance

The accumulative process of evolution, whether its adaptive or nonadaptive, tends to follow paths-of-least-resistance (sensu Anderson et al. 2024). This can be couched in terms of genetic correlations (Arnold and Philips 1999; Chenoweth et al. 2010; McGlothlin et al. 2018) or selection trade-offs (Endler 1983; Shutler 2011; Ercit and Gwynne 2015; Heinen-Kay et al. 2015; Klomp et al. 2016) where the path-of-least-resistance often results in what is essentially a phenotypic middle ground (eg Gerhardt and Brooks 2009; Revell et al. 2010; see also Podos 2022). More generally, though, mechanistic constraints related to physiology and morphology can determine the bounds of phenotypic differentiation (Anderson et al. 2024), and there are scenarios where these constraints can result in unexpected adaptive innovation (see “How constraints foster innovation”). Paths-of-least-resistance also manifest at macroevolutionary scales (eg McGlothlin et al. 2018; Fasanelli et al. 2022; Anderson et al. 2024; Rohner and Berger 2025) and become especially obvious when the disparate evolutionary starting points of distantly-related species result in alternative adaptive solutions (innovations) to the same selection pressure (Ord and Summers 2015; see “Why history matters for innovation”; NB: Anderson et al. 2024 outline how paths-of-least-resistance can explain adaptive convergences in what seem like unusual types of behavior as well).

Mechanistic constraints and disparate starting points can both prompt adaptive innovation separately and synergically. Some examples highlighted in the following sections will be familiar to many behavioral ecologists and communication biologists. However, a fresh look at their mechanistic and phylogenetic context reveals how innovation in behavior can originate and in a manner that has not been widely appreciated. This re-evaluation also emphasizes how a shift of perspective to one that considers the mechanistic and evolutionary factors affecting present-day behavior not only reveals broader interpretations of a behavior—eg instances where the same constraints operating within species have resulted in remarkably different, and currently unrecognized, innovations across species—but also new avenues of study—eg cases where the evolutionary history of species hint at the potential for adaptive innovations that have yet to be formally investigated.

### How constraints foster innovation

Behavioral ecologists are fully aware of the influence of physiology and other mechanistic factors on animal communication. Obvious examples include how mating and aggressive signals function to convey honest cues on a signaler's condition (Clutton-Brock and Albon 1979; FitzGibbon and Fanshawe 1988; Brandt 2003; Sanvito et al. 2007; Rohner and Blanckenhorn 2018; Dougherty 2021), how the production of certain signals is dependent on hormonal triggers or metabolic constraints (Weiss and Moore 2004; Ophir et al. 2010; Mangiamele et al. 2016; Ord and Stamps 2017; Anderson et al. 2021), and how allometry impacts the types of vocalizations, displays or ornaments animals use for communication (Szekely et al. 2004; Kodric-Brown et al. 2006; Friis et al. 2021; Ord et al. 2023). Less appreciated is how these same mechanistic influences can generate diversity, and specifically, innovations in communication across closely-related species.

**Figure 2:**
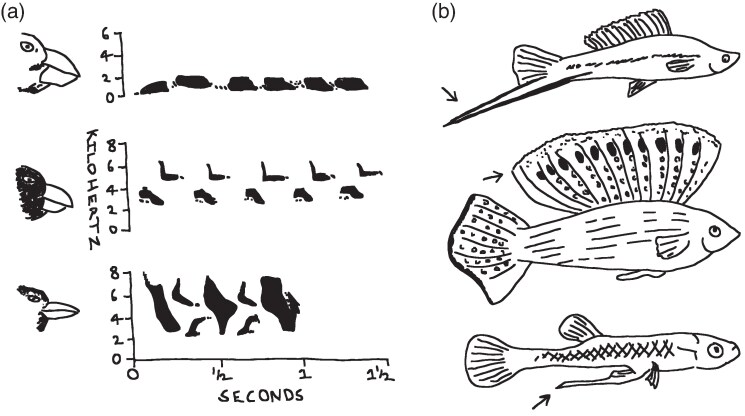
Why mechanistic constraints matter for the evolution of signal innovations. Adaptation in beak size a) for processing different food types by *Geospiza fortis*, *Camarhynchus psittacula* and *Certhidea olivacea* (as examples; here, shown from top to bottom) impact the way beaks convey sound, and this in turn has generated novelties in song across species (Podos 2001). In fishes b), male swordtails (*Xiphophorus maculatus*; Basolo 1990a), sailfin mollies (*Poecilia velifora*; Kozak et al. 2008) and mosquitofish (*Gambusia affinis*; Langerhans et al. 2005) cheat the constraint of developing large body size through signal innovations that tap into female preferences for large mates. The arrows highlight the sword, sailfin and gonopodium, respectively. Drawings are taken from the graphic novel *Understanding Animal Behaviour* (Ord 2025), a free educational resource that can be used to supplement or replace any commercially available textbook on animal behavior (ordlab.unsw.edu.au/understanding-animal-behaviour).

For instance, the sounds animals produce are not only impacted by gross body size, but also the physical structures used to make vocalizations (reviewed by Podos et al. 2004b). This includes the characteristics of vocal organs, like the larynx (Elemans et al. 2024), trachea (Fournier et al. 2024) and syrinx (Suthers and Margoliash 2002), and the shape of the vocal tract more generally (Riede et al. 2006), but also the way the mouth is used to propagate sound. When these physical structures differentiate in response to selection—or alternately remain conserved while species diverge in habitat or behavior—the constraints imposed on sound production can lead to the evolution of novel signal designs. A classic example involves the size and shape of the beak in birds, which is often the result of adaptation to the types of foods eaten (Navalon et al. 2019). Darwin's finches are a particularly strong illustration of this, with the efficiency of processing food (Grant et al. 1976; Schluter and Grant 1984) being a potent selection pressure on beak morphology (Boag and Grant 1981; Grant and Grant 2002). This has produced considerable diversity in beak shape and size across finch species within the Galapagos archipelago (Grant 1991; al-Mosleh et al. 2021). Song is central to contest and mating behavior in these finches (Ratcliffe and Grant 1985; Grant and Grant 1996, 2010; Christensen et al. 2006), and the size and shape of the beak filters the types of sounds birds can include in those songs (eg Nowicki 1987). The constraints imposed by beak morphology have subsequently generated alternative song designs across the finches (Podos 2001; Podos et al. 2004a; Huber and Podos 2006). Species with large, robust beaks adapted for cracking open hard-shelled seeds have songs characterized by low syllable repetition and narrow sound frequences, while those with small, more gracile beaks adapted for catching small insects or processing soft seeds produce songs with many syllables across a variety of sound frequencies (Podos 2001; Fig. 2a).

This same phenomenon of new song designs arising from the mechanical constraints imposed by foraging adaptations to the beak has been demonstrated in other avian systems as well (Derryberry et al. 2012; see also Langin et al. 2017), including driving song divergence within species (Badyaev et al. 2008). While the morphological constraint of a large beak might be viewed as limiting the evolution of song design from the perspective of an individual species (eg Podos et al. 2004b), the range of sounds produced by different beaks across closely-related taxa has clearly generated song diversity (Podos 2001; Badyaev et al. 2008; Derryberry et al. 2012). In this comparative context, and because the function of these songs is equivalent across these species—ie to attract mates and defend territories—the less appreciated outcome is these alternative signal designs represent evolutionary innovations for the same adaptive function rather than cases of evolutionary limitations.

The phenomenon of signal innovation evolving because of a physiological constraint has also occurred in fishes (see also Turner et al. 2007), and in a way that appears not to have been previously realized. Swordtails are an iconic case study of how pre-existing female biases have driven the evolution of an ornamental tail extension in males—the “sword” (Basolo 1990a, 1990b). Controversy surrounded the discovery of this same bias in females of closely-related platyfish (Fig. 2b), where males lacked the sword entirely and females were unlikely to have seen a sworded heterospecific in nature (Basolo 1991, 1995, 1996; da Silva 1991; Winquist et al. 1991; Meyer et al. 1994). Subsequent analyses have continued to support the initial conclusion (Basolo 1990a) that this bias in females predated the evolution of the sword in males (eg Cui et al. 2013). Although there remains some confusion over why this bias in females exists (eg Anderson et al. 2024), empirical evidence for its origin was in fact obtained soon after the controversy initially broke. The bias is not for the sword specifically, but a general preference in females for larger mates (initially proposed by Ryan and Keddy-Hector 1992 and experimental confirmed by Rosenthal and Evans 1998; see also Caves and Kelley 2025 for a related, recent test). Female preference for large males as mates is widespread in nature (eg Gilburn et al. 1992; Ryan and Keddy-Hector 1992; Plath et al. 2007; Poole and Murphy 2007; Deb et al. 2012). For example, this preference can drive the evolution of male-biased sexual size dimorphism in some species (Horne et al. 2020). In swordtails, however, males have innovated a “cheat” through the evolution of an ornament (the sword) that provides the illusion of large size (Rosenthal and Evans 1998) while circumventing the physiological constraints associated with evolving increased body size.

This same cheat manifests in different ornamental innovations in other fishes. Female sailfin mollies exhibit a preference for large males, and males have evolved an exaggerated dorsal fin to appear bigger to females (Kozak et al. 2008; Fig. 2b). It appears mollies could have even evolved the same sword as male swordtails and been just as effective at tapping into female preferences for large mates. In an evolutionary “what if” experiment, Makowicz et al. (2016) used video playback to confirm female sailfin and Tamesi mollies preferred conspecific males digitally appended with a sword to their caudal fin. The large gonopodium of male mosquito fish also seems to cheat female preferences for large mates (Langerhans et al. 2005; Fig. 2b). Note, other signal innovations where males incorporate colors into their ornaments that exploit female biases for certain colored foods (eg the red nuptial colors of male sticklebacks [Smith et al. 2004] and the orange body ornamentation of guppies [Rodd et al. 2002]) are different phenomenon entirely because these innovations are not prompted by signaler constraints on color production. In contrast, the sword in male swordtails, the exaggerated dorsal fin in male mollies and the large gonopodium of male mosquito fish are all alternative signal innovations circumventing the constraints associated with developing large bodies and serve the same function of attracting females during courtship (Rosen and Tucker 1961; Farr et al. 1986; Rosenthal et al. 1996). Although the origins of these exaggerated ornaments are (largely) recognized as signals tapping into a common female preference, it is only once a broader comparative perspective is taken does it become obvious—and seemingly for the first time—of how a mechanistic constraint on the signaler prompted the evolution of these signal innovations.

There has been a somewhat related discussion of the interplay of signaler constraints and receiver perception for explaining the evolution of similarities (rather than innovations) in signal design among taxa (eg Anderson et al. 2024; MacGillavry 2025). Of note is the case presented by Anderson et al. (2024) where certain muscle groups that are particularly sensitive to androgen regulation have predisposed the ritualization of unusual actions into convergent signals among different species, especially actions that also happen to trigger existing sensory biases in receivers. These androgen sensitive muscle groups explain convergences in seemingly peculiar behaviors like foot-flagging frogs or dewlapping lizards (see also “Why history matters for innovation”). That is, these behaviors are not, in fact, unusual at all when the physiological “biases” inherent in signalers and the perceptual biases of receivers are explicitly taken into consideration to reveal the convergent path-of-least-resistance among species (Anderson et al. 2024). Yet as exemplified by swordtails, mollies and mosquito fish, there are mechanistic constraints that also restrict the elaboration of some features (eg body size) in favor of the elaboration of entirely different (not similar) characteristics in different species (eg the caudal fin, dorsal fin and gonopodium, respectively). It is these types of first-order physiological and morphological constraints on signalers that are likely to act as general filters of signal evolution (Fig. 1), and one that promotes evolutionary novelty (rather than convergence).

In a last example to reiterate this point, a ground-breaking investigation into the underlying metabolic basis of acoustic communication hints at numerous potential signal innovations worth further study. Compiling data for hundreds of species across all major taxonomic groups, Gillooly and Ophir (2010) discovered body mass and temperature explained an extraordinary amount of variation in signal behavior among species. As species increase in body mass, and depending on whether species are ectotherms or endotherms, there is a predictable decrease in sound frequency and rate of production, coupled with an increase in the duration and loudness of calls. That is, the types of vocalisations animals produce are largely determined by their mass and body temperature. This is because the size of species and their body temperature mediate energetic and neuromuscular constraints (Gillooly et al. 2001), which in turn bound the types of sounds animals can evolve (Gillooly and Ophir 2010; Ophir et al. 2010). If it was possible to standardize species to the same size and body temperature, the vocalizations used by animals to communicate with one another would sound remarkably alike (ie similar frequency, rate of repetition, duration and loudness).

Yet there were clear outliers in the Gillooly and Ophir data that potentially correspond to some species circumventing the metabolic constraints associated with sound production, which in turn could point to the evolution of signal novelties. These innovations likely reflect alternative adaptive solutions for sound production, but also the evolution of new call types (eg see Podos et al. 2004b for discussion). The evolution of long tracheas allow many bird species to break the constraining link between gross body size and call frequency, such that these birds can produce calls lower in sound frequency than expected for their small size (Fitch 1999). An innovation in the structure of the larynx of humpback whales allows these animals to produce vocalizations at substantially higher sound frequencies than their massive size should allow (Elemans et al. 2024). This has effectively opened the door to the evolution of considerable signal complexity in humpbacks and other whales who have independently evolved the same innovation (Elemans et al. 2024; see Martin et al. 2017 for a broader perspective of the innovations in vocal frequency between marine and terrestrial mammals).

Other prominent outliers in Gillooly and Ophir's data include a diverse group of animals who seem to have adaptively converged on an entirely new signal type, the vocal “trill”. Insects, amphibians, fish, birds and mammals have a variety of species that seem to have independently evolved trills, which consistently exhibit longer durations with fewer repeats than other vocalizing animals (Gillooly and Ophir 2010). This latter call design presumably reflects the performance constraints associated with producing rapid sounds of broad frequency (Podos 1997; Elemans et al. 2004; Sierro et al. 2023; reviewed by Podos et al. 2004b). By extension, the evolution of trills across such disparate taxa could be the outcome of selection for an honest means of assessing signaler quality, which has placed these species onto a new adaptive trajectory of signal design. Birds have been a special focus for investigations into trill evolution (eg Podos 1997; Sierro et al. 2023), but the broader implications of this signal type arising in other major taxonomic groups (eg Naguib 2003; Blankers et al. 2015) remains mostly unexplored.

The Gillooly and Ophir (2010) data offer a rich starting point for identifying species capable of producing sounds that “break” the near-universal metabolic constraints on vocal communication associated with body size and temperature. At the least, morphological modifications in sound producing structures have certainly resulted in innovative solutions in signal design (eg Fitch 1999; Elemans et al. 2004; Elemans et al. 2024), but there are likely to be other innovations yet to be discovered.

There is perhaps an obvious argument here that if some groups have evolved a workaround to a mechanistic constraint, then perhaps it was not a true constraint in the first instance (Podos 2022). This indicates a need for defining constraint beyond simply describing it as some underlying physiological or morphological factor. At a general level, there are various views of what constitutes “constraint”. Some consider the biases of receiver sensory systems as the key constraining filter through which all signal designs must pass (eg Anderson et al. 2024). Others emphasize the physical environment or the evolutionary order of individual signal components as necessary constraining forces that determine what elaborations are more or less likely to evolve (MacGillavry 2025). By extension, early discussions of phylogeny and adaptation effectively presented phylogenetic “effects”, or more specifically the tendency for descendant taxa to retain features from an evolutionary ancestor, as a constraint on the likelihood of adaptation occurring in response to present-day selection pressures (eg Brooks and McLennan 1991; Gittleman and Decker 1994).

However, in the context of the mechanistic constraints discussed in this review, constraint is better considered some inherent aspect of an individual's physiology or morphology that acts as a first-order factor dictating the trajectory of adaptive evolution *before* either environmental or receiver filters come into play (in the specific case of communication; see Fig. 1). The influence of precursor adaptations or the order of historic adaptation on the likelihood of further elaboration is a phenomenon better considered separately (see why history matters for innovation” below). Treating phylogenetic “effects” as a constraint muddles pattern and process because phylogeny neither restricts nor directs evolutionary change (Blomberg and Garland 2002; Losos 2008; Nunn 2011). Podos (2022) provides the best definition of constraint that aligns with the sentiment of its use in my review: any “proximate” factor that limits the capacity of individuals within a given generation to express or develop a characteristic, but where selection could erode or circumvent this limitation over evolutionary time. It in turn implies the importance of considering constraints on animal behavior comparatively across species rather than isolated within a single species, and as a different element from the contingent outcome of the adaptive process.

### Why history matters for innovation

The contingent nature of the adaptive process is a common theme in evolutionary biology, although one that has prompted some controversy on the likelihood of adaptive convergence occurring in nature (reviewed by Ord and Summers 2015). What is not argued is the general tendency of adaptive evolution to build on what has gone before, or what Darwin presented as “descent with modification” (Darwin 1859) and others have called the “recursive” process of evolution (Rossler 1979; Miles et al. 2020) or “historical contingency” (Gould 1989; Ord and Summers 2015). Here, selection is limited to operating on existing species phenotypes, with the specific origin of new features being the general domain of random mutation (eg Blount et al. 2008; Linnen et al. 2013; Wielgoss et al. 2013; Herdegen and Radwan 2015; Houle et al. 2017; see also Mendelson et al. 2014). History matters because the phenotypic characteristics of evolutionary ancestors are the ingredients upon which historic selection works, and the stochastic nature of mutation, genetic drift and other chance events affects how adaptation progressively unfolds over time (Blount et al. 2008, 2018; Campbell et al. 2010; Meyer et al. 2012; Daane et al. 2019; see also the classic critique by Gould and Lewontin 1979). This means adaptive convergence among species experiencing common selection environments is less likely to occur the greater the phylogenetic distance separating species. This is because the disparity in evolutionary starting points coupled with the stochastic nature of the evolution process effectively places lineages on increasingly divergent adaptive trajectories (see analyses reported in Ord and Summers 2015). The end outcome is instead the origin of alternative adaptive solutions or, more specifically, the evolution of signal innovations.

The consequences of such historical contingencies have rarely been a focus in the study of animal communication systems (a point most recently echoed by MacGillavry 2025, although highlighted several decades prior: eg Brooks and McLennan 1991; Ryan and Rand 1993; Martins 1996). Yet, evidence does show the vagaries of evolution can account for the origin of signal innovations. Before elaborating on some examples in nature that have been identified in the context of communication, it is worth mentioning a remarkable simulation by Wischmann et al. (2012) that helps conceptualize how the order of events can shape subsequent evolutionary innovations in behavior. Replicate populations of cooperatively foraging robots were let loose into identical arenas and the evolution of their behavior tracked over time. These arenas had a single “food” source, which a robot could only detect when sitting immediately over it. Each robot was equipped with light-emitting diodes, a neural network and an artificial genome. The robots rapidly evolved a communication system to convey information to one another on the position of the food source by flashing their light diodes. This cooperative behavior took the form of either a 1-color or 2-color signal. Which signal strategy evolved was dependent on the specific order of behaviors arising in a population. This type of mutation-order impact on adaptation, despite starting from identical evolutionary starting points, occurs in an organic setting as well (Wichman et al. 1999; Blount et al. 2008; see also Schluter 2009 and Mendelson et al. 2014). Moreover, mutations can initially arise that have little to no impact on an organism's phenotype and only come into the focus of selection once a later mutation occurs that then interacts with the previous mutation to increase fitness (ie epistasis; Wichman et al. 1999; Blount et al. 2008). Mutation-order essentially magnifies the probability of phenotypic differentiation among lineages despite those lineages being exposed to similar (or even the same) selection pressure. That is, lineages can differentiate adaptively without any divergence occurring in ecology simply because of the order of mutations that arise randomly in different populations (Mendelson et al. 2014).

More generally, the divergent adaptive outcomes resulting from such historical contingencies have been called many-to-one mapping of form to function (Alfaro et al. 2004; Wainwright et al. 2005; Thompson et al. 2017) or functional redundancy (Alfaro et al. 2005; McGee and Wainwright 2013). Examples are of special interest to evolutionary biologists because they highlight how adaptive innovations originate in nature as an outcome of historical starting points and the stochasticity inherent in the evolution process. Many-to-one adaptations, or adaptations that are functionally redundant among taxa, are a special type of convergent adaptation (Alfaro et al. 2004; McGee and Wainwright 2013). When taxa adaptively evolve in response to a common selection pressure, there are 3 potential outcomes positioned along a continuum of form (Ord and Summers 2015). At one end is “parallel evolution” where the same phenotypic form evolves to fill the same functional outcome through common genetic and developmental pathways (eg Zakon et al. 2006; Soria-Carrasco et al. 2014), often (but not always) among closely related taxa (eg independent origins of the same adaptation in separate populations of the same species; Wood et al. 2005; Stern 2013; but see Arendt and Reznick 2008; Stuart 2019). At the other end of the continuum is functionally redundant phenotypes that are explicitly different in form but otherwise fulfill the same functional outcome, often among distantly related taxa (eg alternative adaptive innovations evolving in taxa from different families; Hagman and Ord 2016). Between these 2 extremes sits “classical convergence” where the same phenotypic form evolves to fill the same functional outcome but through different genetic and developmental pathways, often (but not always) among species of the same genus or family (NB: textbook cases of convergence that many of us are probably familiar with—like morphological adaptations allowing powered flight between birds and bats—are so striking because they are otherwise exceptional examples between highly disparate groups, whereas nearly two thirds of reported convergences in the literature typically occur among species within the same family; see Ord and Summers 2015).

By definition, functionally redundant adaptations are cases of alternative evolutionary innovations arising in response to a common selection pressure and are only identifiable through a phylogenetic lens. There are currently few examples of the phenomenon in behavior, but almost certainly because of a lack of research focus rather than a biological absence (Ord and Summers 2015). For instance, strong cases of functionally redundant innovations in communication are apparent in the design and elaboration of territorial displays of lizards, which also provide exemplars of why historical contingency matters for behavioral adaptation more generally.

Male yellow-chinned anoles on the island of Puerto Rico establish and defend territories through the performance of elaborate movements of the head and body, coupled with the extension of a large throat fan called a dewlap (Ord and Stamps 2008). Poor habitat light and visual noise generated by windblown vegetation present an acute challenge to the efficiency of these visual displays (eg Ord and Stamps 2008). To maximize signal transmission, male lizards tailor the timing (Ord et al. 2011), duration (Ord et al. 2015a), speed (Ord et al. 2007, 2015a) and design of displays (Ord and Stamps 2008) on a moment-to-moment basis depending on the prevailing conditions in the surrounding environment. This behavioral flexibility is a form of phenotypic plasticity called “contextual” plasticity (Stamps and Groothuis 2010; also known as “activational” plasticity: Snell-Rood 2013). Other anole species on Puerto Rico from the same phylogenetic radiation exhibit the same signal timing and contextually plastic behavior (Ord et al. 2007, 2010, 2011, 2015a). These behaviors have therefore been retained in present-day species on Puerto Rico from a common evolutionary ancestor. Anoles on the island of Jamaica from a different phylogenetic radiation exhibit none of these behaviors, despite still advertising territory ownership using head-bobs, push-ups and dewlaps in habitats comparable to those on Puerto Rico (Ord et al. 2010, 2011).

**Figure 3:**
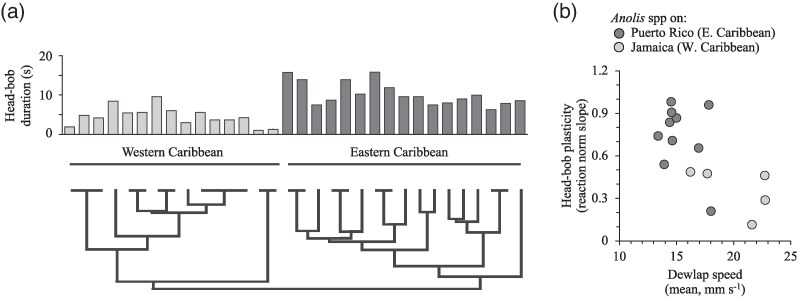
Why historical contingency matters for the evolution of signal innovations. The divergent evolutionary history of *Anolis* lizards from the Western and Eastern Caribbean is reflected in major differences in the design of territorial visual displays (Ord 2012). Species from the Western Caribbean typically perform displays with a) shorter head-bob sequences and b) faster dewlap extensions, compared with the longer, slower displays of the Eastern Caribbean species. In particular, the rapid extension of the dewlap by Western Caribbean anoles (like those on Jamaica) appears to be an effective, and energetically inexpensive, means of maintaining signal efficacy in visually difficult environments (Ord and Stamps 2008; Ord et al. 2013). Eastern Caribbean species, on the other hand, lack the anatomical innovation that allows high speed dewlap movement. Instead, Eastern Caribbean anoles (like those on Puerto Rico) rely on timing display production to avoid visually noisy conditions (Ord et al. 2011), and when they do display, tailor both the speed and duration of the more energetically-expensive head-bob movements to the prevailing conditions in the surrounding environment (Ord et al. 2007, 2015a). Data shown in a) is reported in Ord (2012) and b) in Ord et al. (2010, 2021).

This divergence in behavior appears to extend well beyond these 2 islands to include the broader phylogenetic radiations of the Eastern Caribbean (Puerto Rico, Hispaniola) and the Western Caribbean (Jamaica, Cuba, Bermuda, Cayman Islands and Florida; Ord 2012; Fig. 3a). Jamaican and other Western Caribbean species have apparently resolved the problem of how to communicate effectively in visually difficult environments by concentrating display effort around the rapid extension of the dewlap (Ord et al. 2011; Fig. 3b). Robot playback experiments to free-living anoles have confirmed the rapid and repeated extension of the dewlap (on Jamaica and putatively throughout the Western Caribbean) and plasticly increasing the speed of head-bob and push-up movements (on Puerto Rico and putatively throughout the Eastern Caribbean) are both equally effective strategies for long-range communication in visually noisy conditions (Ord and Stamps 2008; see also Ord et al. 2021). That is, these alternative display strategies are functionally redundant innovations.

Anatomical study across species traces the likely cause of this signal divergence to a change in muscle physiology powering the dewlap extension in the evolutionary ancestor of Western Caribbean anoles (Ord et al. 2013). This historical innovation in muscle physiology appears to be the direct result of selection on signal production (rather than a co-option of a feature initially derived for something else). It allows these anoles to extend the dewlap at far greater speeds than seems to be possible for Eastern Caribbean anoles. This has placed Western and Eastern Caribbean anoles on alternative adaptive trajectories for coping with visually challenging environments: repeated bouts of high-speed movements in 1 signal component, the dewlap, (found in Western Caribbean anoles) versus selective timing of display production and contextual plasticity in multiple signal components (Eastern Caribbean anoles).

**Figure 4:**
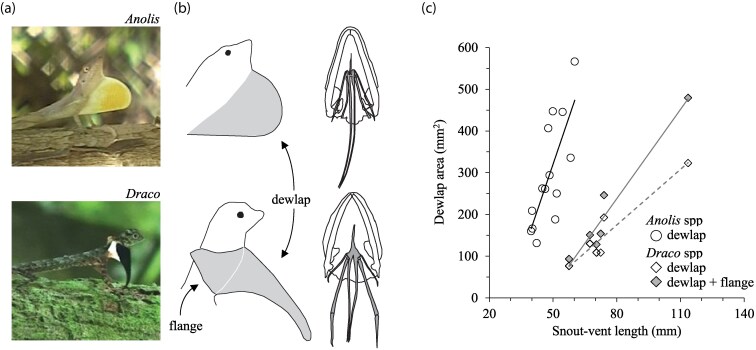
Why historical contingency and mechanistic constraints combined matter for the evolution of signal innovation. The Caribbean *Anolis* and Southeast Asian *Draco* lizards have a) independently evolved an extendible dewlap for territorial display (Ord et al. 2015b; represented here by *Anolis lineatopus* [top panel] and *Draco melanopogon* [bottom panel]). Evolution of the dewlap has occurred through (b) substantial elaborations to the underlying hyoid apparatus (Hagman and Ord 2016). These hyoid elaborations have proceeded very differently in the 2 groups, and in the case of *Draco*, has effectively capped the maximum size the dewlap can achieve. To compensate, *Draco* have evolved b) flanges to the dewlap that function to c) increase the overall area of the visual signal to broadly comparable sizes to those exhibited by *Anolis* (data reported in Summers and Ord 2022).

Taking an even broader phylogenetic perspective, the head-bob and push-up display itself evolved well before the dewlap some 130 to 160 million years ago and has since been retained in most lizards of the iguanid and agamid families (Ord et al. 2023). Remarkably the dewlap has independently evolved at least 3 times: once in iguanid lizards (the anoles; ∼80 million years ago) and twice in agamid lizards (*Sitana-Otocryptis* and *Draco*; both ∼40 to 50 million years ago; Ord et al. 2015b, 2021; Fig. 4a). All 3 groups use headbobs and dewlaps to advertise territorial ownership (Decourcy and Jenssen 1994; Mori and Hikida 1994; Zambre et al. 2020), so the displays are employed in a functionally redundant manner for communication. In at least the agamids, the dewlap seems to have originated as an elaboration of an existing throat ornament to enhance the conspicuousness of head-bob and push-up displays in forest environments (Ord et al. 2015b). The underlying hyoid morphology that controls the extension and retraction of the dewlap is fundamentally different across the 3 lizard groups (Hagman and Ord 2016; Fig. 4b). There appears to be no obvious mechanistic constraint on hyoid adaptation (see also further discussion of the physiological predispositions of the dewlap by Anderson et al. 2024), so the divergent morphologies of the hyoid are likely the consequence of the disparate evolutionary starting points of each group (Ord et al. 2015b). In the case of the Southeastern *Draco* lizards, the way the hypoid apparatus has been elaborated into an extendible dewlap has placed critical constraints on its overall size, which has in turn been compensated for by the evolution of another signal innovation described below.

Dewlap size is an important aspect of the conspicuousness of the dewlap in visually difficult environments (Losos and Chu 1998; Ord et al. 2013; Klomp et al 2016). Across both Puerto Rican and Jamaican anoles, dewlap size increases with declining habitat light (Summers and Ord 2022) presumably because larger dewlaps are easier to spot in dimly lit environments (Losos and Chu 1998). However, the unique way the hyoid has been elaborated in southeastern Asian *Draco* lizards has effectively capped its gross size (Summers and Ord 2022): species with larger dewlaps are prone to scraping the dewlap along the substrate as its extended (eg see Fig. 4a). In what appears to be an innovation in morphological design, *Draco* have evolved neck flanges that are extended at the same time as the main dewlap (Hagman and Ord 2016; Summers and Ord 2022). These increase the overall area of the signal to achieve comparable dewlap sizes to those of Caribbean anoles (Summers and Ord 2022; Fig. 4c). The comparison of the dewlap between Caribbean anoles and southeast Asian *Draco* offers a striking illustration of many-to-one mapping of form to function (ie functional redundant adaptation; Hagman and Ord 2016). It showcases how historical contingency and mechanistic constraints interact to generate alternative signal innovations between distantly related species that otherwise communicate for the same function (to advertise territory ownership) in comparable habitats (Ord et al. 2021).

Furthermore, in an evolutionary scenario reminiscent of the adaptive shift in signal design towards the rapid, repeated extension of the dewlap in Jamaican and Western Caribbean anoles (Ord 2012), there are a subset of *Draco* from the Malay radiation who have lost the head-bob and push-up entirely from their signal repertoire (Ord et al. 2023). This subset of species instead relies entirely on the extension of the dewlap for communication (Ord et al. 2023). This concentration of signal effort exclusively to the dewlap appears to have occurred because of the increased energetic costs associated with performing high-speed head-bob and push-ups displays in species with unusually large body sizes (Ord et al. 2023). The loss in information potential encoded by head-bob and push-up displays (eg, Nelson et al. 2022) has been mitigated to some degree through the evolutionary innovation of greater color complexity of the dewlap (Ord et al. 2023).

If cases of classical convergence in animal communication are rare (see Introduction), functionally redundant forms of communication are even harder to come by in the literature (Ord and Summers 2015). Nevertheless, there are clear examples in birds where divergent historical starting points have generated extensive diversity in signal design across species (Miles and Fuxjager 2019; Miles et al. 2020; MacGillavry 2025).

Take the example of the woodpeckers (Miles et al. 2020). These birds drum by hitting their bills repeatedly against a hard surface (eg tree trunk) as a territorial defence signal (Schuppe and Fuxjager 2018). A history of range overlap between some congeners has resulted in rhythm or cadence differentiation in drumming (Miles et al. 2020). This differentiation presumably results from agonistic character displacement (Grether et al. 2009) to ensure adequate species identity cues for discriminating between species that frequently come into contact. However, the manner in which sympatric species have diverged in cadence has in turn dictated how lineages subsequently elaborate the speed and duration of drumming in response to sexual selection (Miles et al. 2020). The combined outcome of past selection for species recognition between species, followed by sexual selection within species, is extensive diversity in drumming pattern across the woodpecker group. Much of this diversity reflects alternative innovations in the cadence, speed or duration of drumming for the functionally redundant outcome of effective territorial defence. Yet, in some species, the initial divergence in drum cadence in sympatry has restricted elaboration of the drum and despite strong sexual selection still operating within those species. These particular woodpecker species offer an unexplored opportunity for future research into the evolution of alternative innovations in vocalization and plumage that compensate for restrictions on drum elaboration (see Discussion by Miles et al. 2020).

Although potentially unaware of the broader phenomenon of many-to-one or functionally redundant adaptation, MacGillavry (2025) reveals several other cases in the birds of paradise of what appears to be exactly that. Males of 4 bird of paradise genera perform courtship displays involving a mesmerizing black shapeshifting dance that morphs a male's body into something truly otherworldly (readers are encouraged to see images presented in MacGillavry (2025) or search online for videos of the courtship of *Parotia sefilata*, *Epimachus fastuosus*, *Lophorina niedda* and *Ptiloris magnificus*). The feathers and body movements used in these displays differ between the 4 genera, but from the perspective of an inspecting female the outcome would presumably be strikingly similar. Regardless, these male shapeshifting displays are clearly functionally equivalent across species as a courtship ritual for enticing females to mate. Although yet to be formally investigated as products of adaptive (functional) convergence, MacGillavry (2025) suggests the disparate evolutionary starting points of each genera, coupled with an interaction between low-light environments and receiver perceptual biases that are shared across these genera, is likely responsible for the (functionally redundant) convergence of these displays.

## When the sum is greater than the parts

Tinbergen (1963) advocated animal behavior could only be properly understood through investigation of function (“survival value”), mechanism (“causation”), development (“ontogeny”) and evolution (“phylogeny”). While behavioral ecology and ethology has had its fads of research foci over the intervening decades (Durant 1986; Dawkins 1989; Dewsbury 1992; Alcock 2003; Huntingford 2003; Ord et al. 2005; Guadarrama-Maillot and Waas 2008), as well as debates over the utility of Tinbergen's paradigm (Alcock and Sherman 1994; Bateson and Laland 2013; Nesse 2013), it seems fair to say animal behavior as an overarching field of study is one of the most integrative of the life sciences. Given this, there are probably few readers who would not be familiar with how the mechanistic basis (or more broadly proximate factors) of behavior determine its expression, or why going beyond single-species study is vital for not only identifying the similarities and differences in species behavior across groups, but how behavior has likely evolved (eg Lorenz (1941) was one of the first to do this). What is currently underrepresented in our research, however, is the explicit study of how mechanistic constraints lead to evolutionary paths-of-least-resistance and how historical contingency sets evolutionary starting points, both of which can independently or synergistically generate innovations in behavior to common selection pressures (Fig. 1; see also Anderson et al. (2024) and MacGillavry (2025) for additional discussion).

Darwin's (1859) initial description of natural selection as descent with modification should be a reminder that present-day adaptation inherently carries the baggage of evolutionary history (or “ghosts of biases past”: Ryan and Rand 1993). The increasing availability of published phylogenies and publicly available data sets herald new opportunities for studying the evolutionary origins of behavior. Yet the implicit starting assumption of many studies is present-day behavior is the product of contemporary adaptation: eg differences in signal design across closely related species presumably reflect differences in the conditions experienced by those species in their respective habitats. When evolutionary history has been considered, it is often a statistical correction of phylogenetic relationships. This follows Felsenstein's (1985) observation that species cannot be treated as independent data points because phenotypes will likely be more similar among closely related taxa because of shared ancestry. This was a critical point to make at a time when across-species comparisons were beginning to gain traction in evolutionary ecology (eg Wyles et al. 1983; Cheverud et al. 1985; Ryan and Brenowitz 1985; Sillen-Tullberg 1988). However, the field of phylogenetic comparative methods has moved on substantially since this classic paper (Felsenstein 1985). Statistical methods are now vastly more sophisticated and can explicitly consider the historically contingent nature of the evolution process in a variety of forms (readers are referred to Miles and Fuxjager (2019) and Miles et al. (2020) as particularly good practical illustrations of some of these modern approaches).

Embracing modern phylogenetic comparative methods and reframing research questions to consider phenomena such as functional redundancy will not only open new avenues of research on signal evolution, but offer an alternative lens through which to evaluate what we think we already know about how behavior evolves. Unexplored cases of probable innovation include species who seem to have circumvented the metabolic constraints associated with the production of vocalisations revealed by Gillooly and Ophir (2010), or the woodpeckers identified by Miles et al. (2020) who appear constrained in elaborating their drumming, so presumably have compensated through innovations in vocalization or plumage. Previously unrecognized cases of alternative adaptive solutions to common selection pressures start to become apparent when viewed through a phylogenetic lens of many-to-one or functional redundancy. These include iconic cases of song constraint resulting from beak morphology in birds (Podos 2001) or seemingly unrelated ornaments evolving in fishes (Basolo 1990a; Langerhans et al. 2005; Kozak et al. 2008).

There have been many shifts in how we view the origins of animal behavior over the last 50 years or so. The role of female mate choice in sexual selection was initially controversial, but is now a common theme underlying much research (Kirkpatrick 1987; Andersson 1994; Andersson and Simmons 2006; Clutton-Brock 2007; Rosenthal and Ryan 2022). Concepts of receiver psychology (Guilford and Dawkins 1991), sensory bias (Ryan and Keddy-Hector 1992; Basolo and Endler 1995), and sensory drive (Endler 1992) were hot topics in the 1990s and are now central to our understanding of signal evolution (Miller and Bee 2012; Rowe 2013; Cummings and Endler 2018; Renoult and Mendelson 2019; Caves et al. 2025). Yet, at a general level, animal behavior is no different to the rest of an animal's phenotype. Factors that bound the adaptive trajectories of morphology, physiology and development also bound the evolution of behavior. Phenomena like historical contingency and functional redundancy should therefore be common place in our vernacular of behavioral evolution.

There have been recent calls by Anderson et al. (2024) and MacGillavry (2025) to better integrate mechanistic constraints and receiver biases, or historical contingencies and receiver biases, into the study of adaptive convergence in signal design. Many communication biologists would probably feel a receiver's view of animal signals is reasonably well met in most contemporary research programs (eg see references in previous paragraph). The arguments of Anderson et al. (2024) and MacGillavry (2025) therefore rest more squarely on the need for better representation of mechanistic constraints and historical contingencies in current research. The intention of my review is to bring constraint and contingency more explicitly together to illustrate how their consideration fills a potentially hidden gap in our current knowledge of why diversity exists in nature (Fig. 1). Constraint and contingency not only manifest in functional convergences in behavior—interesting in its own right because of what it reveals about alternative adaptive solutions to common selection challenges (eg Figures 2b and 4)—but also how diversity in behavior among species might not always be attributable to differences in present-day selection among species (eg Figures 2a and 3). Instead, mechanistic constraint and historical contingency act as first-order filters that subsequently corral the trajectory of signal evolution before contemporary selective pressures (like receiver biases) necessarily come into play (Fig. 1).

Given that animal behavior dictates how species interact with the world around them, which in turn determines the types of selection pressures acting on species, it's a little odd that evolutionary ecology is not dominated by behavioral ecologists (eg most evolutionary ecology research centers on morphology; Ord and Summers 2015; see also Stamps 2003). Nonetheless, the perspectives of constraint and contingency can be drawn from evolutionary biology to provide new opportunities for identifying and understanding innovations in behavior. By recognizing the contingent nature of evolution and considering the underlying constraints that shape behavior, our understanding of how diversity in the natural world arises will be greatly expanded.

## Data Availability

There are no original data associated with this review. Data shown in Figs. 3 and 4 are reported in Ord (2012); Ord et al. (2010, 2021) and Summers and Ord (2022).
